# Cardiovascular magnetic resonance findings in Danon disease: a case series of a family

**DOI:** 10.3389/fcvm.2023.1159576

**Published:** 2023-05-04

**Authors:** Xiaolong Liu, Ning Zhai, Xiaoqiang Wang, Jiehuan Wang, Mengchun Jiang, Zhanguo Sun, Yueqin Chen, Jingjing Xu, Yinghua Cui, Lu Li

**Affiliations:** ^1^ Department of Radiology, Affiliated Hospital of Jining Medical University, Jining, China; ^2^ Department of Cardiology, Affiliated Hospital of Jining Medical University, Jining, China; ^3^ Department of Pathology, Affiliated Hospital of Jining Medical University, Jining, China

**Keywords:** Danon disease, glycogen storage disease, LAMP2, cardiovascular magnetic resonance, late gadolinium enhancement, feature tracking

## Abstract

**Background:**

Cardiac involvement constitutes the primary cause of mortality in patients with Danon disease (DD). This study aimed to explore the cardiac magnetic resonance (CMR) features and progressions of DD cardiomyopathies in a family with long-term follow-up.

**Methods:**

Seven patients (five females and two males), belonging to the same family and afflicted with DD, were enrolled in this study between 2017 and 2022. The cardiac structure, function, strain, tissue characteristics on CMR and their evolutions during follow-up were analyzed.

**Results:**

Three young female patients (3/7, 42.86%) exhibited normal cardiac morphology. Four patients (4/7, 57.14%) displayed left ventricle hypertrophy (LVH), and mostly with septal thickening (3/4, 75%). A single male case (1/7, 14.3%) showed decreased LV ejection fraction (LVEF). Nonetheless, the global LV strain of the four adult patients decreased in different degree. The global strain of adolescent male patients was decreased compared to the age-appropriate female patients. Five patients (5/7, 71.43%) exhibited late gadolinium enhancement (LGE), with proportion ranging from 31.6% to 59.7% (median value 42.7%). The most common LGE location was the LV free wall (5/5, 100%), followed by right ventricle insertion points (4/5, 80%) and intraventricular septum (2/5, 40%). Segmental radial strain (*r_s _*= −0.586), circumferential strain (*r* = 0.589), and longitudinal strain (*r* = 0.514) were all moderately correlated with the LGE proportions of corresponding segments (*P *< 0.001). T2 hyperintense and perfusion defect foci were identified, overlapping with the LGE areas. During follow-up, both the young male patients exhibited notable deterioration of their cardiac symptoms and CMR. The LVEF and strain decreased, and the extent of LGE increased year by year. One patient underwent T1 mapping examination. The native T1 value was sensitively elevated even in regions without LGE.

**Conclusions:**

Left ventricular hypertrophy, LGE with sparing or relatively less involved IVS, and LV dysfunction are prominent CMR features of Danon cardiomyopathy. Strain and T1 mapping may have advantages in detecting early-stage dysfunction and myocardial abnormalities in DD patients, respectively. Multi-parametric CMR can serve as an optimal instrument for detecting DD cardiomyopathies.

## Introduction

Danon disease (DD) (OMIM#300257) is a rare X-linked dominant lysosomal glycogen storage disorder induced by lysosome-associated membrane protein-2 (LAMP-2) deficiency (1). Dozens of LAMP-2 mutations have been revealed thus far. The systemic accumulation of glycogen primarily affects organs such as the heart and skeletal muscle. Clinically, it is characterized by a triad of cardiac abnormalities, skeletal myopathy, and varied intellectual disabilities. Cardiac involvements including hypertrophy cardiomyopathy (HCM), dilated cardiomyopathy (DCM), Wolffe-Parkinsone-White (WPW) syndrome, and ventricular arrhythmias, are the leading cause of death in patient with DD (2). Among male DD patients who have not received cardiac transplantation, death before the age of 25 is prevalent (2, 3). The prognosis for female DD patients is relatively better than for males and is primarily dependent on the severity of cardiac disease (4, 5). Early diagnosis is crucial for predicting the course of DD, diagnosing family members to establish appropriate medical follow-up, and preventing transmission to subsequent generations. Genetic testing is the gold standard for diagnosis. Nevertheless, both initial screening and assessment of cardiac involvement largely rely on non-invasive cardiac imaging techniques.

As is well known, cardiac magnetic resonance (CMR) is well-established for accurately evaluating cardiac structure, function, and tissue characteristics in various types of cardiomyopathies (6). Most notably, late gadolinium enhancement (LGE) has an unparalleled advantage in displaying focal fibrosis *in vivo* (7, 8). The utility of CMR in DD has been demonstrated in a few previous studies (9–12). A high prevalence of left ventricular hypertrophy (LVH), LGE of the left ventricle (LV), and reduced LV ejection fraction (LVEF) has been reported. However, due to the rarity of DD all over the world, prior research has mainly focused on case or series reports with small patient populations. Most of these patients came from different families with different mutation types, and did not undergo CMR follow-up. Consequently, comprehensive information about CMR features and follow-up changes in large cohorts of patients with the same mutation types remains limited. In this study, we present seven patients with DD from the same family with long-term follow-up to elucidate the main features of DD, specifically focusing on its CMR findings and evolutions.

## Materials and methods

### Patients

The proband experienced palpitations following conscious activity since age 13. He was initially admitted to our hospital at 14 years old due to palpitations accompanied by transient loss of consciousness. Echocardiography showed that the thickness of left ventricular (LV) posterior wall and interventricular septum (IVS) were 15 mm and 14 mm, respectively. LVEF remained within the normal range (65%). Additionally, electrocardiogram (ECG) demonstrated WPW syndrome. Subsequently, Danon disease was diagnosed at Beijing Union Hospital through electron microscopic examinations and genetic testing (13).

In 2017, his family underwent clinical and genetic evaluation at our hospital. Clinical data and related examination results were collected from electronic patient records, and follow-up continued until July 2022. For deceased family members, information was obtained through a medical history survey. The study protocol was approved by the institutional review board. Signed inform consent was obtained from all study participants.

### CMR protocol

All CMR examinations were conducted on 3.0 T MRI scanners (MAGNETOM Verio, Siemens, Erlangen, Germany, or Ingenia CX, Philips Healthcare, Best, The Netherlands) with eight-channel or thirty-two-channel coil using retrospective ECG gating.

Cine cardiac images were performed by breath-hold steady-state free precession (SSFP) in short-axis (from base to apex), four-chamber, two-chamber, and left ventricle outflow tract (LVOT) views. T2-weighted short-tau inversion recovery (T2w-STIR) images were obtained in multiple short-axis views with an 8 mm slice thickness, encompassing the entire LV and four-chamber views. First-pass perfusion imaging was acquired along with the administration of a bolus intravenous injection of 0.05 mmol/kg gadolinium-DTPA (Magnevist, Bayer Healthcare, Berlin, Germany) at 4 ml/s through an arm vein. LGE images were obtained 10–15 min after intravenous administration of another 0.15 mmol/kg gadolinium-DTPA in multiple short-axis (from base to apex), two- and four-chamber views with phase-sensitive inversion recovery sequence. Native and post-contrast T1 mapping were obtained before and 15 min after the administration of gadolinium-DTPA, respectively, by a modified Look-Locker inversion recovery (MOLLI) method (14).

### CMR analysis

CMR data were analyzed on an offline workstation using commercially available software (CVI42, 5.13.5 version, Circle Cardiovascular Imaging, Calgary, Alberta, Canada). All the images were measured and assessed through consensus by two cardio-radiologist experts who were blinded to the clinical data. Any inconsistency was resolved by a senior supervisor.

Segmental end-diastolic LV wall thickness (LVWT) was measured using short-axis cine images in a 16-segment American Heart Association (AHA) model. Moreover, the right ventricular (RV) wall thickness, end-diastolic dimension of LV (LVEDd) and RV (RVEDd) were measured. LV phenotype, asymmetric hypertrophy, and symmetric hypertrophy were defined according to the previous literature (15, 16). RV hypertrophy was defined as a maximal RV free wall thickness exceeding 5 mm (10). The short-axis cine stack was used to calculate the LV end-systolic volume, LV end-diastolic volume, LV ejection fraction (LVEF), cardiac output, and LV mass. Meanwhile, the body surface area was employed to index the volume and mass data. Global and segmental LV strain was computed via CMR feature-tracking technique from short-axis, two- and four-chamber cine images, and the reference range was determined according to the previous study (17).

To avoid any influence on the judgment, T2-weighted images and perfusion images were assessed prior to LGE images. Hyperintense T2-weighted signal would be recognized if the ratio of myocardial signal intensity to skeletal muscle was greater than 1.9 (10). Perfusion defect was defined as a regional decrease in myocardial signal during the LV first-pass of contrast agent, unrelated to artifacts (18). LGE of the LV was recorded on a 17-segment AHA model (including the apex), and RV insertion points (RVIP) was assigned to proximal intraventricular septum (IVS) segment with additional markings. LGE patterns were categorized into four types according to their distribution from endocardium to epicardium: subendocardial, mid-mural, subepicardial, and transmural (when more than 50% of the myocardial thickness was involved) (19). Global and segmental LGE proportions (LGE mass/LV mass) were acquired by setting a signal intensity threshold at 5 standard deviations above the mean intensity of a reference region with no visible LGE (9, 20) in multiple short-axis views using 16-segment AHA model. The presence of LGE of the RV free wall, if any, was also recorded. Native T1 maps were automatically calculated, and subsequently the global and segmental native T1 values (the mean, minimum, and maximum) were acquired. Extracellular volume fraction (ECV) was calculated by using the previously-established equation: myocardial ECV = (1-hematocrit)×(*δ*R1_myocardium_/*δ*R1_blood_), where R1 = 1/T1, and hematocrit level was determined from a venous blood sample prior to the CMR examination within 24 h. Then we obtained the maximal and minimal ECV by drawing areas of interest in regions with and without LGE, respectively.

### Data analysis

Data analysis was performed by SPSS software (version 25.0, IBM, Armonk, New York). Normality was determined using the Shapiro–Wilk statistical test. Continuous variables were presented as median and ranges. Categorical variables were presented as frequency and proportion. The correlation between segmental LVWT, LV strain and LGE proportions were assessed using Pearson's correlation coefficient (for jointly normally distributed data) and Spearman's correlation coefficient (for non-normally distributed data). All tests were two-sided, and *P*-values less than 0.05 were considered statistically significant.

## Result

### Study population and the baseline clinical feature in 2017

**Figure 1:**
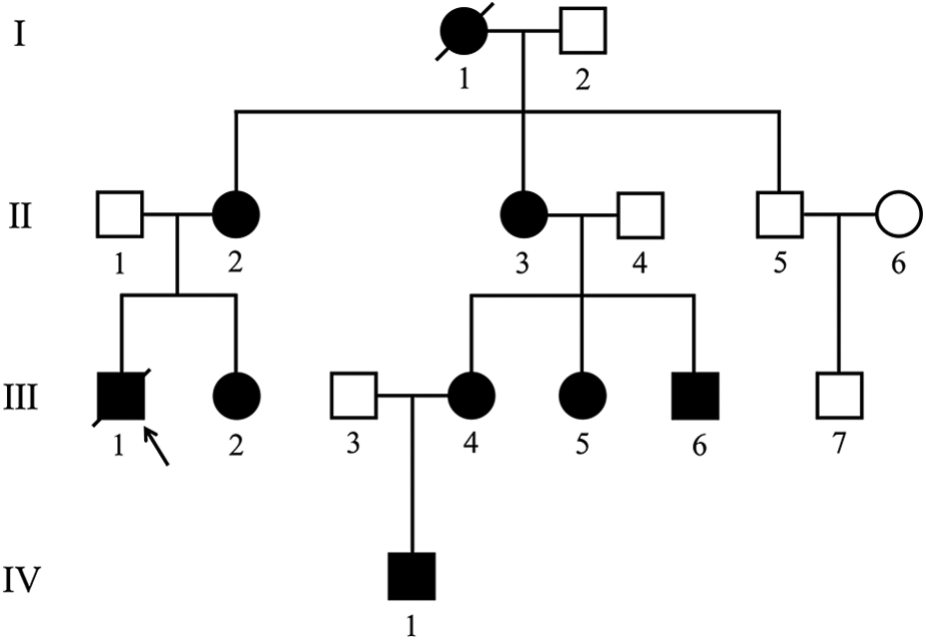
Pedigree of the family with Danon disease. Circles, female individuals; squares, male individuals; slashes symbols, deceased individuals; black shapes, affected subjects; white shapes, unaffected subjects; arrow, proband. I-1 died of heart disease at the age of 45. Ⅲ-1 suddenly died of heart disease at 22.

By summarizing the genetic analysis and medical history, the pedigree of the family was obtained (Figure 1). Among the fourteen family members from generation Ⅱ–Ⅳ, eight individuals [including the proband (Ⅲ-1)] were confirmed to have frameshift mutation secondary to a 2-bp deletion (c.257_258delCC) in the exon 3 of the LAMP2 gene.

CMR examination of Ⅳ-1, an asymptomatic 8-year-old boy, was failed due to lack of cooperation and subsequently excluded from the study. Therefore, seven patients (Ⅱ-2, Ⅱ-3, Ⅲ-1, Ⅲ-2, Ⅲ-4, Ⅲ-5, and Ⅲ-6) performed CMR examinations were eventually enrolled, including five females (median age 23 years old, range from 14 to 44 years old) and two males (14 and 20 years old). Their demographic features, clinical manifestations, ECG and laboratory results are summarized in Table 1.

**Table 1:** Baseline clinical features, ECG, and lab results in 2017.

Case No.	Sex	Age in 2017	Onset age	Symptoms	Intellectual disabilities	ECG	AST (U/L)	ALT (U/L)	CK (U/l)	CK-MB (ng/ml)	LDH (U/L)	HBDH (U/L)	Treatment	Age at death
Ⅱ-2	F	44	30	Palpitation, dyspnea	Normal	Inverted T wave	28	14.7	86	16	309	271	Medicine	Alive
Ⅱ-3	F	41	30	Palpitation, dyspnea	Normal	Inverted T wave	25	10.4	95	20	303	276	Medicine	Alive
Ⅲ-1	M	20	13	Palpitation, dyspnea, syncope	Attention deficit and learning difficulty	WPW	296	288.1	1,960	47	1,259	1,283	Medicine	22
Ⅲ-2	F	15	N/A	Fatigue	Normal	WPW	41	12.4	146	15	359	379	Medicine	Alive
Ⅲ-4	F	23	21	Palpitation	Normal	Inverted T wave	39	31.7	130	19	306	346	Medicine	Alive
Ⅲ-5	F	14	N/A	Fatigue	Normal	Inverted T wave	50	13.9	138	25	369	397	Medicine	Alive
Ⅲ-6	M	14	13	Fatigue	Learning difficulty	WPW	398	338.9	1,986	42	1,551	1,080	Medicine	Alive

F, female; M, male; ECG, electrocardiogram; WPW, Wolffe parkinsone white syndrome; N/A, not available; AST, aspartate aminotransferase; CK, creatine kinase; CK-MB, creatine kinase isoenzyme- MB; HBDH, α-hydroxybutyrate dehydrogenase; LDH, lactate dehydrogenase; normal reference values: ALT (11–66) U/L, AST (15–46) U/L, CK (2–200) U/L, CK-MB (0–25) ng/ml, LDH (94–250) U/L, HBDH (80–220) U/L.

Both IV-1 and IV-6 experienced cardiomyopathy with earlier onset during their second decade of life. Ⅱ-2, Ⅱ-3, and Ⅲ-2 manifest cardiomyopathy during adulthood. Four patients presented with palpitation (Ⅱ-2, Ⅱ-3, Ⅲ-4, and Ⅲ-1), three with dyspnea (Ⅱ-2, Ⅱ-3, and Ⅲ-1), three with fatigue (Ⅲ-2, Ⅲ-5, and Ⅲ-6), and one with syncope (Ⅲ-1). Moreover, Ⅲ-1 exhibited attention deficit and learning difficulty, and Ⅲ-6 displayed learning difficulty. During the 12-lead ECG examination, Ⅲ-2, Ⅲ-1 and Ⅲ-6 were accompanied by WPW syndrome. Additionally, ECG of Ⅱ-2, Ⅱ-3, Ⅲ-4, and Ⅲ-5 showed inverted T waves.

Serum enzyme analyses, including aspartate aminotransferase (AST), creatine kinase (CK), creatine kinase isoenzyme-MB (CK-MB), α-hydroxybutyrate dehydrogenase (HBDH) and lactate dehydrogenase (LDH) were performed. In Ⅲ-1 and Ⅲ-6, the levels of all aforementioned markers were significantly elevated. In the five female patients, only HBDH and LDH levels were slightly elevated.

### Baseline cardiac structure characteristics on CMR in 2017

**Figure 2:**
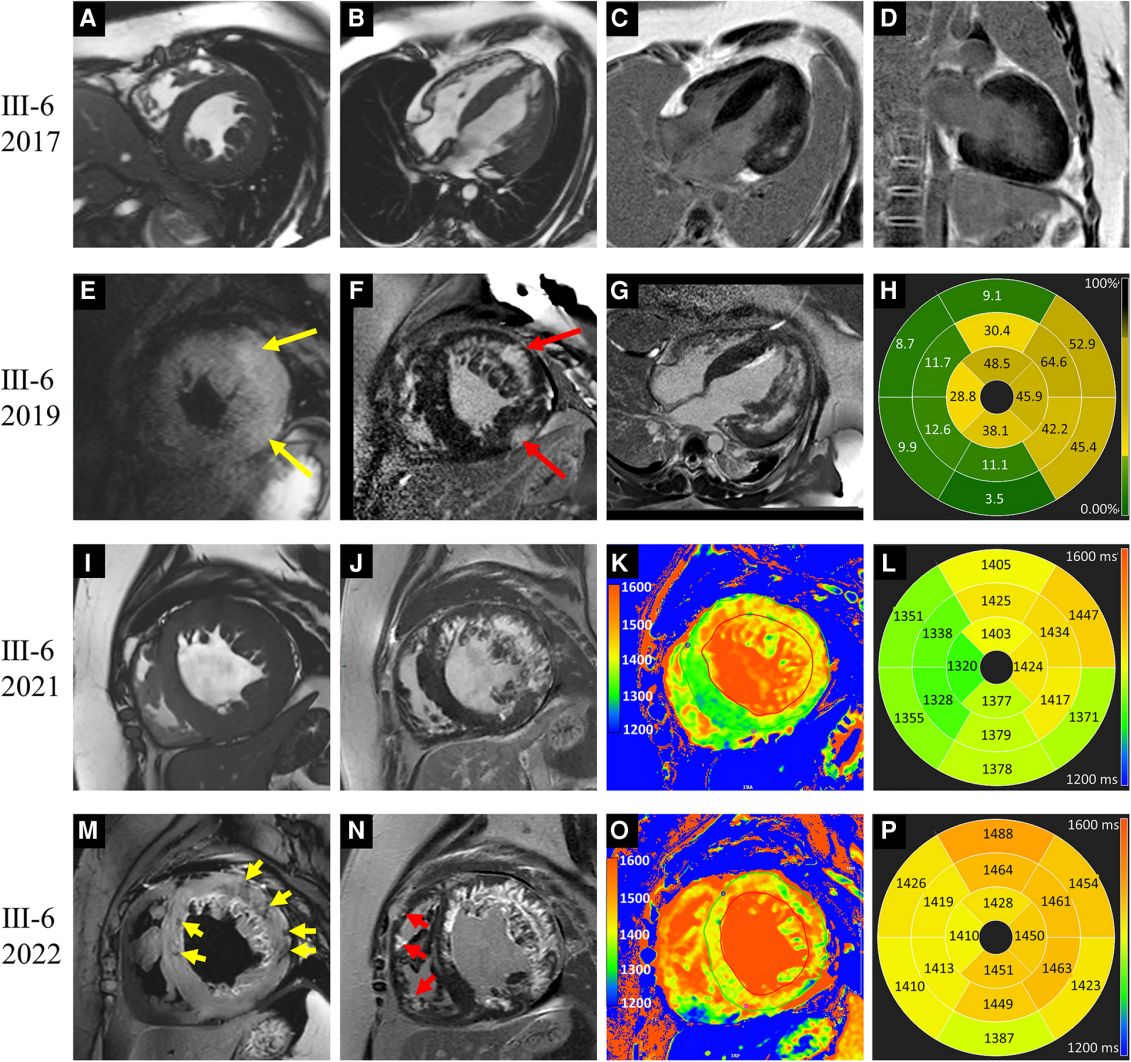
Images of representative patient with Danon disease (Ⅲ-6, a 14-year-old male). In 2017, cine cardiac images revealed diffusely thickened left ventricle (LV) wall (**A,B**). Patchy late gadolinium enhancement (LGE) involved the LV lateral wall (**C,D**). In 2019, hyperintense T2 signal foci (yellow arrows in **E**) were revealed and corresponding to the LGE areas (red arrows in **F**). The extent of LGE was increased and predominantly present in the LV free wall (**G**). The bull's-eye view of LGE proportion showed the apical involvement (**H**). In 2021, LV dilation was detected on cine cardiac image (**I**). T1 value elevated significantly in the LGE areas, and slightly in regions without LGE (**J–L**). In 2022, hypointense T2 signal foci were emerged (yellow arrows in **M**). The LGE extent was increased with the involvement of the intraventricular septum and right ventricle (red arrows in **N**). Moreover, the elevation of T1 value was aggravated (**O,P**).

**Figure 3:**
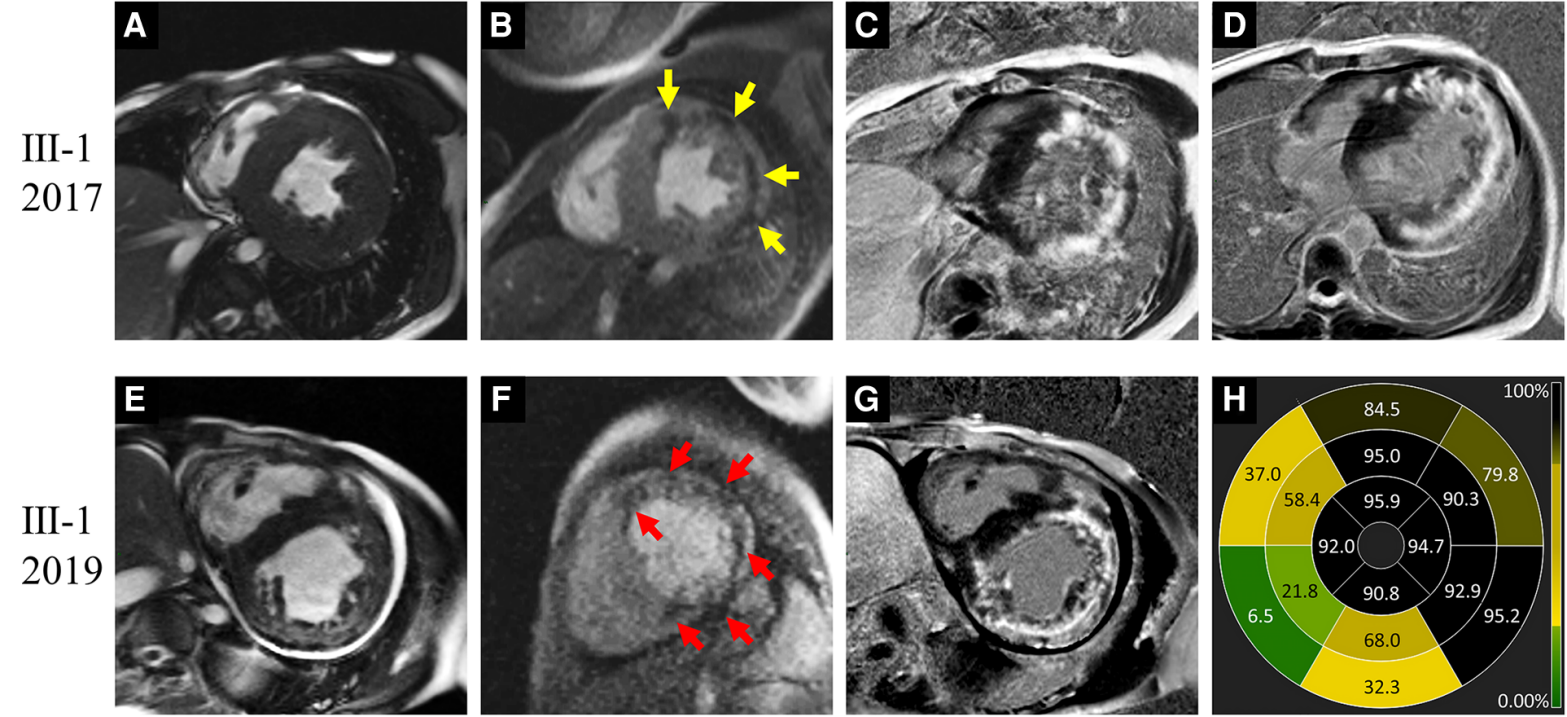
Images of representative patient with Danon disease (Ⅲ-1, a 20-year-old male). In 2017, cine cardiac image showed symmetrically increased thickness of left ventricle (LV) wall (**A**). Resting perfusion defect foci (yellow arrows in **B**) were revealed corresponding to the late gadolinium enhancement (LGE) areas (**C,D**), which predominantly present in the LV free wall and right ventricle insertion points. In 2019, LV dilation was detected on cine cardiac image (**E**). Both the perfusion defect (red arrows in **F**) and LGE foci (**G**) were increased. The bull's-eye view of LGE proportion showed the severe involvement in LV free wall and apex (**H**).

**Figure 4:**
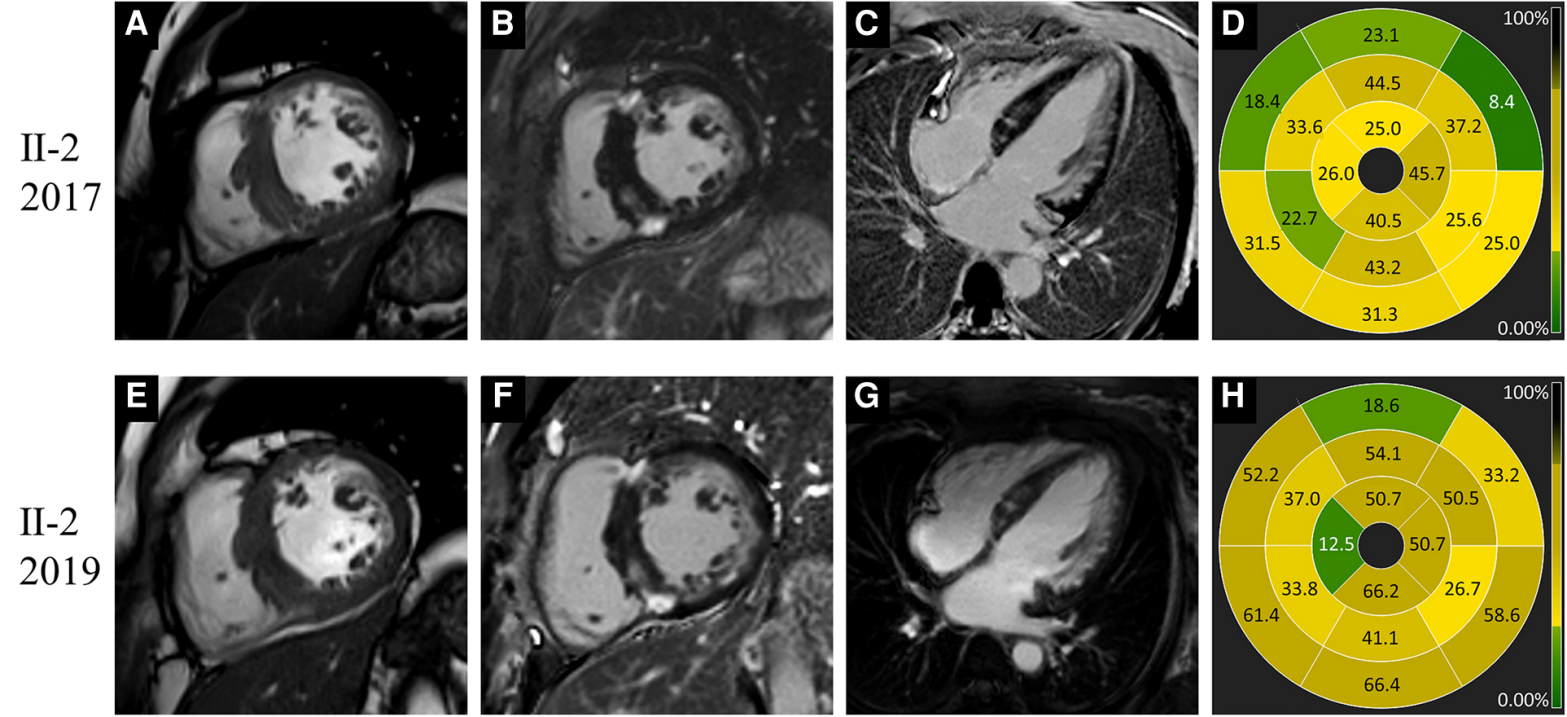
Images of representative patient with Danon disease (Ⅱ-2, a 44-year-old female). In 2017, cine cardiac image in short-axis view showed increased thickness of intraventricular septum (**A**). Late gadolinium enhancement predominantly involved the right ventricle insertion points, the left ventricle free wall and intraventricular septum (**B–D**). In 2019, the change of cardiac structure was slight on cine image (**E**). The extent of late gadolinium enhancement (**F–H**) showed mild to moderate progression compared to 2017.

The cardiac structural features of DD are summarized in Table 2. Three young female patients (3/7, 42.86%) showed normal cardiac morphology (Ⅲ-2, Ⅲ-4, and Ⅲ-5). Four patients (4/7, 57.14%) presented with HCM phenotype, including two with symmetric patterns (Ⅲ-1 and Ⅲ-6; Figures 2A, 3A) and two with asymmetric patterns (Ⅱ-2 and Ⅱ-3; Figure 4A). The maximal LV walls thickness ranged from 16.5 mm to 27.2 mm (median 21.4 mm), predominantly located in the IVS (3/4, 75%), followed by the LV free wall (1/4, 25%). LVOT obstruction was not observed. Except for one case with slight enlargement of LVEDd (Ⅲ-1), the LVEDd in other patients were all within the normal range. Moreover, the RVEDd and RVWT of the seven patients were all within the normal range.

**Table 2:** Cardiac structure and tissue characteristics of Danon disease on CMR and their evolutions during follow-up.

Case No. and Time		Sex/Age (Y)	Phenotype	LVEDd (mm)	LVWT_max_ (mm)	RVWT_max_ (mm)	LVMI (g/m^2^)	T2WI	Perfusion defect	LGE proportion (%)	LGE location	LGE pattern
Ⅱ-2	2017	F/44	Asymmetric HCM	47.2	17.6, IVS	2.8	56.5	Hyper-	Inferior RVIP	35.2	RVIPs + IVS + basal free wall	Subendocardial, mid-mural, and transmural
	2019	F/46	Asymmetric HCM	46.2	18.3, IVS	3	58.7	Hyper-	Inferior RVIP	44.1	RVIPs + IVS + basal free wall	Subendocardial, mid-mural, and transmural
Ⅱ-3	2017	F/41	Asymmetric HCM	50.0	16.53, IVS	2.5	63.7	Hyper-	RVIPs + LV free wall	42.7	RVIPs + LV free wall + basal IVS	Mid-mural and transmural
Ⅲ-1	2017	M/20	Symmetric HCM	55.6	27.2, IVS	3.5	71.2	Hyper-	RVIPs + LV free wall + basal IVS	59.7	RVIPs + LV free wall	Subendocardial, mid-mural, and transmural
	2019	M/22	Symmetric HCM	64.5	24.2, IVS	7.1	76.8	Hyper-	RVIPs + LV free wall + basal IVS	77.8	RVIPs + LV free wall + basal IVS	Subendocardial, mid-mural, and transmural
Ⅲ-2	2017	F/15	Normal	43.5	11.4, IVS	2.2	52.9	Normal	Normal	/	/	/
Ⅲ-4	2017	F/23	Normal	43.3	11.3, IVS	2.4	51.2	Hyper-	Inferior RVIP + LV free wall	46.3	RVIPs + LV free wall	Subendocardial and mid-mural
Ⅲ-5	2017	F/14	Normal	45.3	10.3, IVS	2.4	48.9	Normal	Normal	/	/	/
Ⅲ-6	2017	M/14	Symmetric HCM	53.0	26.3, LV free wall	3.4	139.2	Hyper-	LV free wall	31.6	LV free wall	Mid-mural
	2019	M/16	Symmetric HCM	63.2	32.8, LV free wall	5.3	189.2	Hyper-	LV free wall	54	LV free wall + IVS	Subendocardial, mid-mural, and transmural
	2021	M/18	Symmetric HCM	72.0	26.58, LV free wall	6.6	174.6	Hyper-and hypo-	LV free wall + IVS	59.4	LV free wall + IVS + RV	Subendocardial, mid-mural, and transmural
	2022	M/19	Symmetric HCM	74.5	25.3, IVS	7.3	165.	Hyper-and hypo-	LV free wall + IVS	66.2	LV free wall + IVS + RV	Subendocardial, mid-mural, and transmural

F, female; M, male; HCM, hypertrophic cardiomyopathy; LVEDd, left ventricle end-diastolic dimension; LVWT_max_, maximal LV wall thickness; IVS, intraventricular septum; RV, right ventricle; LVMI, LV mass index; RVIP, RV insertion point; T2WI, T2 weighted image; LGE, late gadolinium enhancement.

### Baseline cardiac function and strain characteristics on CMR in 2017

Except for one case with decreased LVEF (Ⅲ-1, 34.23%), LVEF in the remaining six cases was normal (Table 3). Global radial strain (GRS), global circumferential strain (GCS), and global longitudinal strain (GLS) were all significantly diminished in Ⅲ-1, and slightly diminished in Ⅱ-3. GRS and GCS of Ⅱ-2 and GLS of Ⅲ-4 were slightly diminished. As for the adolescent patients, GLS, GCS, and GRS of the male patient (Ⅲ-6) were all decreased compared to the female patients (Ⅲ-2 and Ⅲ-5) of his age.

**Table 3:** Function and strains of LV on CMR and their evolutions during follow-up.

Case no. and time		Sex/age (Y)	LVEF (%)	EDVI (ml/m^2^)	ESVI (ml/m^2^)	CI (L/min/m^2^)	GRS (%)	GCS (%)	GLS (%)
Ⅱ-2	2017	F/44	60.43	78.33	31.00	2.98	27.16	−16.7	−19.35
	2019	F/46	49.89	75.94	38.05	2.23	22.65	−14.9	−11.18
Ⅱ-3	2017	F/41	51.02	101.06	49.49	2.84	24.76	−15.9	−15.59
Ⅲ-1	2017	M/20	34.23	110.62	72.75	1.90	9.63	−7.03	−4.52
	2019	M/22	8.03	127.57	117.33	0.72	3.84	−3.3	−2.31
Ⅲ-2	2017	F/15	65.63	76.51	26.30	3.21	34.58	−19.9	−15.89
Ⅲ-4	2017	F/23	60.92	76.61	29.94	3.08	27.46	−16.6	−11.98
Ⅲ-5	2017	F/14	57.67	69.57	29.45	2.73	30.56	−18.4	−13.63
Ⅲ-6	2017	M/14	54.12	89.73	41.17	3.37	21.08	−12.2	−10.88
	2019	M/16	45.15	130.29	71.46	3.39	10.97	−7.69	−4.95
	2021	M/18	24.37	139.40	105.42	2.45	8.24	−6.64	−3.45
	2022	M/19	18.65	162.38	132.10	1.97	6.68	−5.33	−2.71

F, female; M, male; LVEF, left ventricle ejection fraction; EDVI, end-diastolic volume index; ESVI, end-systolic volume indexed; CI, cardiac index; GRS, global radial strain; GCS, global circumferential strain; GLS, global longitudinal strain.

### Baseline cardiac tissue characteristics on CMR in 2017

The cardiac tissue characteristics of DD are summarized in Table 2. Five patients (5/7, 71.43%) exhibited LGE (Figures 2–4), including four patients with HCM phenotype and one patient with normal cardiac morphology (Ⅲ-4). The LGE proportion of these five patients ranged from 31.6% to 59.7% (median value 42.7%). The most common LGE location was LV free wall (5/5, 100%), followed by RVIP (4/5, 80%) and IVS (2/5, 40%). From the AHA segments perspective, there were 69 LGE segments in a total of 85 segments (69/85, 81.18%), with segments 5, 6, 12 and 15 being the most susceptible. Each segment from base to apex may be involved. With regard to the distribution of endocardium-to-epicardium direction, 31 LGE segments (31/69, 44.93%) exhibited transmural involvement, 25 (25/69, 36.23%) with mid-mural involvement, and 13 (13/69, 18.84%) with subendocardial involvement. No LGE was detected in the RV free wall.

Mild to moderate hyperintensity on T2w-STIR were revealed in all five patients with LGE and all emerged in the LGE areas. Likewise, among the five patients with LGE, perfusion defect foci were demonstrated in some areas with LGE (Figure 3B). Regarding the two patients without LGE, no abnormal signal on T2w-STIR and perfusion images were detected.

### Correlation between segmental LVWT, LV strain and LGE proportions

**Figure 5:**
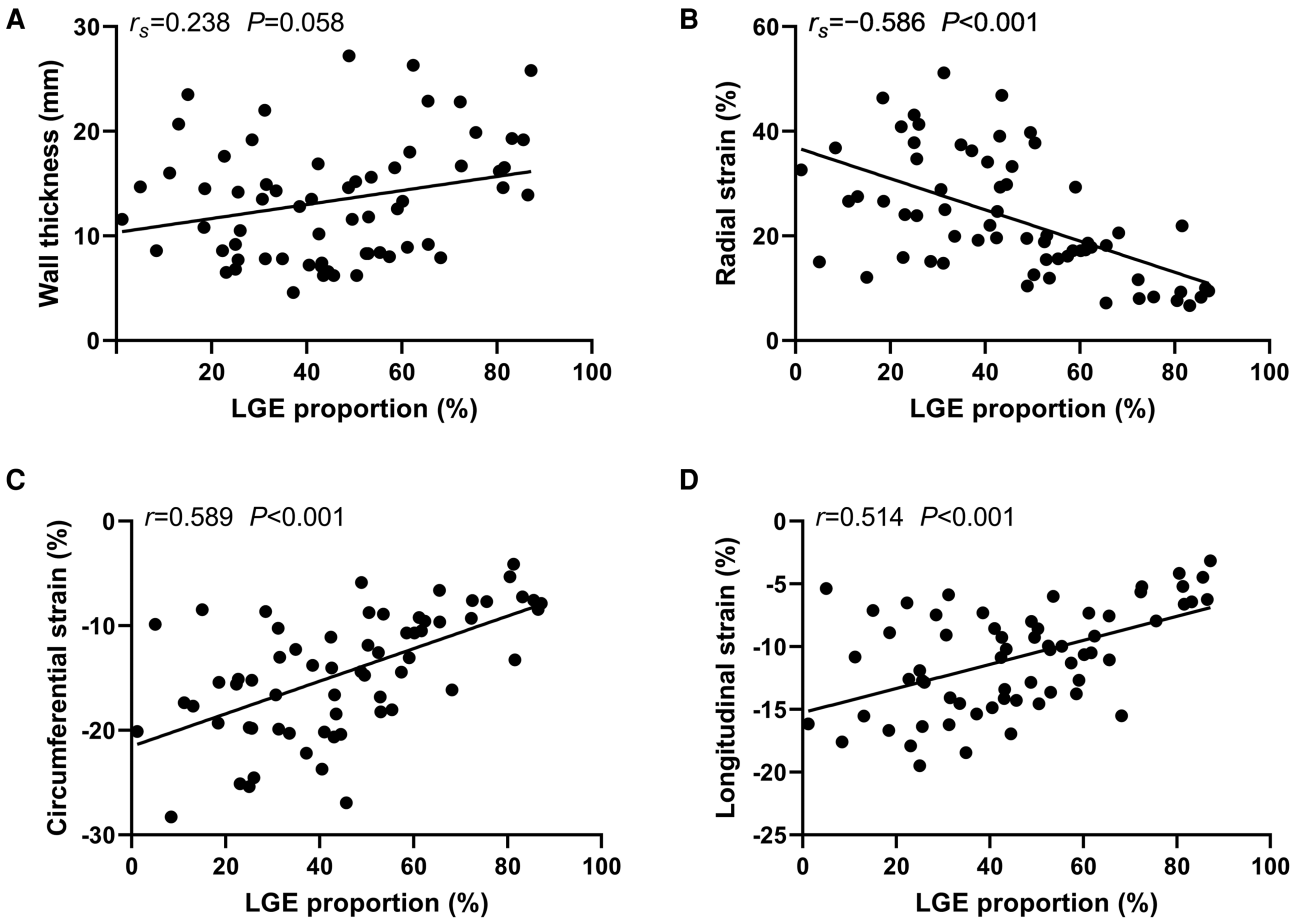
Correlation between segmental left ventricle wall thinckness, strain and late gadolinium enhancement (LGE) proportions. Correlation between the segmental left ventricle wall thinckness and the corresponding segmental LGE proportions (**A**). Correlation between the segmental LGE proportions and the corresponding radial strain (**B**). Correlation between the segmental LGE proportions and the corresponding circumferential strain (**C**). Correlation between the segmental LGE proportions and the corresponding longitudinal strain (**D**). *r_s_*, Spearman's *r*; *r*, Pearson's *r.*

Segmental LVWT, LV strain and LGE proportions were adopted for correlation analysis. There was no significant correlation between the segmental LVWT and the corresponding segmental LGE proportions (Spearman's *r* = 0.238, *P* = 0.058). However, segmental radial strain (Spearman's *r* = −0.586), circumferential strain (Pearson's *r* = 0.589), and longitudinal strain (Pearson's *r* = 0.514) were all moderately correlated with the LGE proportions of corresponding segments (*P* < 0.001) (Figure 5).

### Follow-up and outcome

Follow-up for all the seven patients lasted from 2017 to May 2022. Three patients (Ⅱ-2, Ⅲ-1 and Ⅲ-6) underwent follow-up CMR and summarized in Tables 2, 3.

Patient Ⅲ-6, a male adolescent, experienced multiple hospitalizations due to acute heart failure exacerbation. Cardioverter defibrillator implantation was indicated, but refused by the patient and his parents. Follow-up CMR were performed in 2019, 2021 and 2022 (Figure 2). The LVEF and strains deteriorated over time. LV dilated gradually with an increased LVEDd (74.5 mm in 2022). The LVWT reached its peak in 2019 and then decreased consistently, whereas the RV free wall thickness increased gradually. The extent of LGE in Ⅲ-6 expanded with the involvement of the IVS and RV free wall. Furthermore, significant low T2 signal foci were detected (Figure 2M). In 2021 and 2022, native T1 and ECV were added into the CMR protocol. The global mean, minimum, and maximum T1 values were 1,388, 1,311, and 1,541 ms respectively in 2021; and 1,439, 1,350, and 1,661 ms in 2022. The minimal and maximal ECV were 28% and 72% respectively in 2021; 33% and 86% in 2022. Regarding the regions without LGE, only native T1 values were elevated in 2021, and both native T1 values ECV were elevated in 2022.

Regarding patient Ⅲ-1, a young male, the most prominent CMR change was the decrease of LVEF and strain values in 2019 compared to 2017 (Figure 3). The LV wall slightly thinned, while the RV free wall significantly thickened, with moderately increased LVEDd (64.5 mm) and RVEDd (36.6 mm). The extent of LGE expanded, with the new involvement of the IVS, apex, and RV free wall. Additionally, significant low T2 signal foci emerged within the LGE areas. Cardioverter defibrillator implantation was recommended, but refused by the patient. Unfortunately, he succumbed to heart failure at the age of 22 in 2019.

None of the five female patients (Ⅱ-2, Ⅱ-3, Ⅲ-2, Ⅲ-4, and Ⅲ-5) exhibited substantial symptom deterioration. With regard to Ⅱ-2, the extent and proportion of LGE markedly increased, the LV wall slightly thickened, and the LVEF mildly decreased in 2019 (Figure 4).

## Discussion

This study presents the clinical and CMR features of seven DD patients followed up since 2017. To the best of our knowledge, this is the first study to assess the comprehensive CMR characteristics and evolutions of DD in a large family with long term follow-up. In our cohort, young male patients suffer from DD with severe presentation and aggressive progression vs. female patients. Furthermore, cardiac involvements are the most prominent clinical feature, such as arrhythmias and heart failure. The typically CMR features of DD were LVH, LGE of the LV, diminished strain (global and regional), and preserved or reduced LVEF. Moreover, both T2 hyperintensity and resting perfusion defect were also frequently observed. Consistent with the clinical characteristics, these CMR changes in male patients appeared earlier and more significant, and progressed faster than female patients. As males have one X chromosome, while females have two. The haploinsufficiency of the X-linked LAMP2 gene in male patients, and the degree of skewed X inactivation in females lead to the sex-specific differences in clinical and CMR characteristics (4).

Three young female patients showed normal cardiac morphology. This in part may reflect the late-onset of CMR change in female patients for both of the young males of similar ages presented with profound LVH. In our cohort, LVH in female patients tended to be asymmetric phenotype with predominantly thickened IVS, echoing the previous studies (10, 21). According to previous literatures, DCM may be revealed in affected females (21). Nevertheless, no DCM phenotype was found in our cohort. During follow-up, both of the male patients (Ⅲ-1 and Ⅲ-6) exhibited dilated LV. Considering the persistent LVH, we classified them as “burned-out” HCM phenotype rather than DCM. In the end-stage DD, the thickened LV wall become thinner gradually, accompanied by the LV dilation. Furthermore, asymmetric HCM phenotype may convert to symmetric HCM phenotype or even DCM phenotype at last. Therefore, although LVH is the most common morphological manifestation of DD, patients of different genders and disease stages can exhibit various morphologies.

The global longitudinal, radial, and circumferential peak strain of LV in the adult patients were all decreased to varying degrees. However, LVEF was reduced in only one young male patient. Additionally, the global strain of the adolescent male patient (Ⅲ-6) was decreased compared to the age-appropriate female patients. The high incidence and premature onset of global strain diminution indicate that DD may impact the ventricular contraction and relaxation resulting in a heart failure with preserved ejection fraction in initial stage. This advantage of global strain over LVEF has also been confirmed in previous studies on other heart disease (22, 23). During follow-up, the LVEF and global strain significantly decreased in male patients and slightly decreased in female patients. In comparison to typical HCM, the present male DD patients demonstrated more prematurely, rapidly and significantly reduced LVEF and global strain, which can be used for differential diagnosis of both conditions (5).

LGE is nearly always seen in affected patients. A controlled CMR and histopathological study revealed that LGE regions corresponded closely to the distribution and extent of fibrosis and scarring in Danon cardiomyopathy (24). In our cohort, five patients associated with LGE, including four with HCM phenotype and one with normal cardiac morphology. Hence, LGE may appreciate before morphological changes. Consistent with previous literature, LGE predominantly involve the LV free wall and the RVIPs, rather than the IVS between the anterior and posterior RVIPs (9, 10, 25). Furthermore, most of the LGE areas exhibit transmural or intramyocardial distributions. The patterns of LGE in DD are unlike those in typical HCM, amyloidosis, and sarcoidosis and help in the differential diagnosis (26, 27). During follow-up, LGE of IVS and RV free wall may be demonstrated with disease progression and fibrosis development (28). The degree of LGE corresponds to the risk of cardiac events in multiple types of cardiomyopathies (29, 30). Likewise, LGE may also be applicable to the risk stratification of DD. For example, depending on the LGE proportion of LV and whether the RV is involved. However, a multicenter study with a large sample is still needed for further confirmation.

Native T1 and ECV are predominant quantitative parameters for CMR myocardial tissue characteristics, serving as optimal instruments for detecting diffuse and early-stage myocardial abnormalities. To our knowledge, there are only two previous literatures involving native T1 and ECV alterations in DD (9, 31). In the present study, both native T1 values and ECV significantly elevated in LGE regions, and increased with progression of the condition during follow-up. Regarding the follow-up CMR for patient Ⅲ-6 performed in 2021, native T1 values were sensitively elevated in regions without LGE, while ECV was in normal range. This phenomenon was also seen in a previous study (31). The T1 mapping pattern of DD with elevated T1 values and normal ECV are similar to those of other intracellular storage disorders (32). However, this change is stage-specific, since both T1 values and ECV increase as the aggravation of interstitial and replacement fibrosis.

Our study further investigated the correlation between the segmental LVWT, LV strain and LGE proportions. The results suggest the correlation between the segmental LVWT and the corresponding segmental LGE proportions was not significant (*r_s_* = 0.238, *P* = 0.058). However, in a previous study concerning typical HCM, the regional LGE proportion moderately correlated with the corresponding LVWT (*r* = 0.36, *P* < 0.001) (33). One possible reason is that the distribution of the LVH and LGE in DD cardiomyopathies is relatively mismatched, while both the LVH and LGE are predominantly located in the IVS and the RVIPs in typical HCM (7). Regarding segmental LV strain, segmental radial strain (*r_s_* = −0.586), circumferential strain (*r* = 0.589), and longitudinal strain (*r* = 0.514) were all moderately correlated with the LGE proportions of corresponding segments (*P *< 0.001). These findings suggest that the segmental radial strain value, as well as the absolute values of circumferential and longitudinal strains are negatively correlated with the extent of segmental LGE. Moreover, the global strain is diminished in the stage of heart failure with preserved ejection fraction in DD as revealed in this study. Therefore, it can be speculated that fibrosis and scarring in Danon cardiomyopathy may directly affect the regional LV strain, then the global strain, and eventually the LVEF.

T2w-STIR imaging is one of the best approaches for oedema-weighted CMR, as it suppresses the signal of fat and flowing blood, and enhances sensitivity to myocardial free water content (12, 34). The presence of T2 hyperintensity in Danon cardiomyopathy may represent focal oedema accompanying inflammation and/or ischemia, and reflect an active rather than healed process (34). Notably, low T2 signal foci emerged within the LGE areas in the follow-up CMR of patient IV-1 and IV-6. Low T2 signal suggests excessive fibrosis with minimal free water or even coagulative necrosis and can be considered as a late-stage change (10). In addition, five patients showed resting perfusion defect, with all defect areas coinciding with LGE areas. These perfusion defects are presumed to represent the diminished microvascular bed volume in regions of pronounced fibrosis. Hence, akin to low T2 signal, defected resting perfusion may be used to reflect the extent of fibrosis (20).

There are several limitations to the present study. Firstly, a mere seven cases were incorporated. However, this in part reflects the relatively low prevalence of DD in the general population. It is exceedingly rare for all the seven patients come from the same family. Secondly, follow-up CMR were not conducted for all participants. The CMR follow-up of female patients needs to be further supplemented in. Thirdly, only a single patient underwent quantitative CMR, including T1 mapping and ECV.

## Conclusion

Left ventricular hypertrophy, LGE with sparing involved IVS, and LV dysfunction are common CMR features of Danon cardiomyopathy. LGE of IVS and RV free wall may emerge with the disease progression. Both abnormal T2 signal and perfusion defect foci are not rare, and may be employed to reflect the severity of the lesion. LV strain and T1 mapping may have advantages in detecting early-stage dysfunction and myocardial abnormalities, respectively, in DD patients. Therefore, further investigation is warranted to explore the applications of quantitative CMR techniques in DD.

## Data Availability

The original contributions presented in the study are included in the article, further inquiries can be directed to the corresponding authors.
