# Corrigendum: Cardiovascular magnetic resonance findings in Danon disease: a case series of a family

**DOI:** 10.3389/fcvm.2024.1405171

**Published:** 2024-04-29

**Authors:** Xiaolong Liu, Ning Zhai, Xiaoqiang Wang, Jiehuan Wang, Mengchun Jiang, Zhanguo Sun, Yueqin Chen, Jingjing Xu, Yinghua Cui, Lu Li

**Affiliations:** ^1^ Department of Radiology, Affiliated Hospital of Jining Medical University, Jining, China; ^2^ Department of Cardiology, Affiliated Hospital of Jining Medical University, Jining, China; ^3^ Department of Pathology, Affiliated Hospital of Jining Medical University, Jining, China

**Keywords:** Danon disease, glycogen storage disease, LAMP2, cardiovascular magnetic resonance, late gadolinium enhancement, feature tracking

A Corrigendum on Cardiovascular magnetic resonance findings in Danon disease: a case series of a family By Liu X, Zhai N, Wang X, Wang J, Jiang M, Sun Z, Chen Y, Xu J, Cui Y and Li L (2023). Front. Cardiovasc. Med. 10:1159576. doi: 10.3389/fcvm.2023.1159576

In the published article, there was an error in Table 3 as published. During the plotting of this table, there were incorrect correspondences between the headings of the CI, GRS and GCS columns and the contents below. The contents of the CI column were the GRS values, the contents of the GRS column were the GCS values, and the contents of the GCS column were the CI values. The correspondences between the data in these three columns and the column headings are realigned. The corrected Table 3 and its caption appear below.

**Table 3:** Function and strains of LV on CMR and their evolutions during follow-up.

Case no. and time		Sex/age (Y)	LVEF (%)	EDVI (ml/m^2^)	ESVI (ml/m^2^)	CI (L/min/m^2^)	GRS (%)	GCS (%)	GLS (%)
Ⅱ-2	2017	F/44	60.43	78.33	31.00	2.98	27.16	−16.7	−19.35
	2019	F/46	49.89	75.94	38.05	2.23	22.65	−14.9	−11.18
Ⅱ-3	2017	F/41	51.02	101.06	49.49	2.84	24.76	−15.9	−15.59
Ⅲ-1	2017	M/20	34.23	110.62	72.75	1.90	9.63	−7.03	−4.52
	2019	M/22	8.03	127.57	117.33	0.72	3.84	−3.3	−2.31
Ⅲ-2	2017	F/15	65.63	76.51	26.30	3.21	34.58	−19.9	−15.89
Ⅲ-4	2017	F/23	60.92	76.61	29.94	3.08	27.46	−16.6	−11.98
Ⅲ-5	2017	F/14	57.67	69.57	29.45	2.73	30.56	−18.4	−13.63
Ⅲ-6	2017	M/14	54.12	89.73	41.17	3.37	21.08	−12.2	−10.88
	2019	M/16	45.15	130.29	71.46	3.39	10.97	−7.69	−4.95
	2021	M/18	24.37	139.40	105.42	2.45	8.24	−6.64	−3.45
	2022	M/19	18.65	162.38	132.10	1.97	6.68	−5.33	−2.71

F, female; M, male; LVEF, left ventricle ejection fraction; EDVI, end-diastolic volume index; ESVI, end-systolic volume indexed; CI, cardiac index; GLS, global longitudinal strain; GCS, global circumferential strain; GRS, global radial strain.

The authors apologize for this error and state that this does not change the scientific conclusions of the article in any way. The original article has been updated.

